# Downregulation of miR-125b promotes resistance of glioma cells to TRAIL through overexpression of Tafazzin which is a mitochondrial protein

**DOI:** 10.18632/aging.101939

**Published:** 2019-05-05

**Authors:** Wenjia Ma, Yan Cui, Min Liu, Zhigang Tan, Yugang Jiang

**Affiliations:** 1 Department of Neurosurgery, The Second Xiangya Hospital, Central South University, Changsha 410011, Hu’nan Province, China

**Keywords:** glioma, TRAIL, resistance, miR-125b, TAZ, aging, age-related disease

## Abstract

Overexpression of Tafazzin (TAZ), a mitochondrial protein, is often observed in many cancers. However, the association between aberrant expression of TAZ and drug resistance remains unclear. The aim of this study is to explore the role of TAZ in regulating the TRAIL resistance in glioma. We thus established the TRAIL resistance models on glioma by using the U87 and U251 cell lines (U87/R and U251/R). As the results, obvious overexpression of TAZ was observed in U87/R and U251/R cells. However, knockdown of TAZ increased the sensitivity of U87/R and U251/R cells to TRAIL-induced apoptosis. By contrast, expression of miR-125b was downregulated in U87/R and U251/R cells compared to the parental U87 and U251 cells. Furthermore, decrease of miR-125b was responsible for overexpression of TAZ, because the results of dual-luciferase reporter assays verified that TAZ was targeted by miR-125b. We then showed that enforced expression of miR-125b resensitized the U87/R and U251/R cells to TRAIL-dependent damage of mitochondria and activation of caspase-9 and -3. We demonstrated that overexpression of TAZ caused by downregulation of miR-125b promoted resistance of glioma cells to TRAIL. MiR-125b/TAZ axis may represent a potential strategy to reverse the TRAIL in glioma.

## INTRODUCTION

Glioma is a commonly diagnosed malignant cancer that show poor prognosis and low survival rate. For the advanced glioma patients, systemic chemotherapy is indispensable [1–3]. However, drug resistance is still a major problem in the chemotherapy of various cancers, including glioma [4, 5]. It is urgent to take strategies to overcome the chemoresistance of glioma.

TNF-related apoptosis-inducing ligand (TRAIL) belongs to the member of the TNF superfamily. It triggers caspase-8 and thus induces extrinsic and intrinsic apoptosis. TRAIL-caused extrinsic apoptosis is induced by activation of caspase-8 directly, whereas the intrinsic apoptosis pathway is required the mediation of mitochondria damage [6, 7]. TRAIL has been proved to show high selectivity on cancers. It induces apoptosis of cancer cells without attacking the normal cells. Thus, TRAIL is a low-toxic and promising drug for treatment of cancers. However, some cancer cells, including glioma, usually develop tolerance to TRAIL-induced apoptosis [8, 9]. Therefore, elucidation of the mechanisms of TRAIL resistance and searching strategies to improve the anti-tumor activity of TRAIL are important in the TRAIL-based treatment.

Tafazzin (TAZ) functions as a mitochondria-related protein that localized in the mitochondrial membrane. As TAZ plays a critical role in the remodeling of cardiolipin which is a major lipid in the mitochondrial membrane, TAZ is important to keep the stabilization of mitochondrial membrane potential [10, 11]. Recently, studies have reported that TAZ is overexpressed in several cancers such as rectal cancer, colon cancer and thyroid neoplasms [12, 13]. However, role of TAZ in affecting the resistance of glioma to TRAIL is still required to be explored. MicroRNAs (miRNAs) are endogenous, small and non-coding RNA molecules that regulate approximately 60% of human genes [14, 15]. In the present study, we showed significant upregulation of TAZ in TRAIL-resistant glioma cells. Furthermore, we demonstrated that the downregulation of miR-125b was responsible for the overexpression of TAZ in glioma.

## RESULTS

### TAZ is upregulated in TRAIL-resistant glioma cells

**Figure 1:**
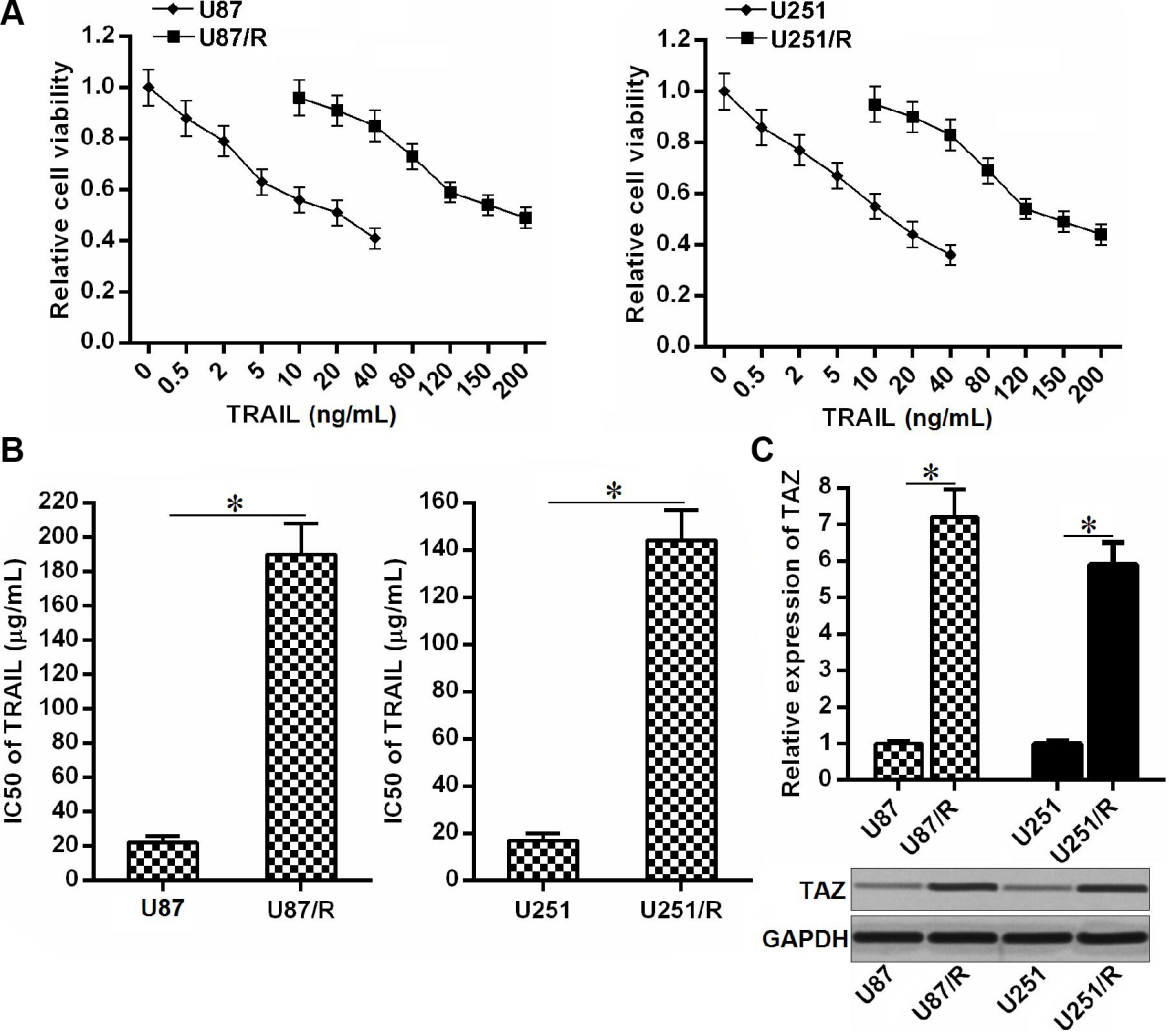
**Overexpression of TAZ in TRAIL-resistant glioma cells.** (**A**) Drug sensitivity of U251, U251/R, U87 and U87/R cells to TRAIL (0~200 ng/mL). (**B**) IC50 of TRAIL to U251, U251/R, U87 and U87/R cells. *P<0.05. (**C**) QRT-PCR and western blot analysis were performed to evaluate the expression of TAZ at the mRNA level and protein level, respectively. *P<0.05.

To study the TRAIL resistance in glioma, we established the TRAIL-resistant U87 and U251 cell lines (U87/R and U251/R) through gradual exposure to increasing concentrations of TRAIL. Results of MTT assays confirmed the low drug sensitivity of U87/R and U251/R cells to TRAIL (Figure 1A). We showed that IC50 of TRAIL to U87/R was 7.67 fold higher than its parental U87 cell line. Meanwhile, IC50 of TRAIL to U251/R was 6.95 fold higher than its parental U251 cell line (Figure 1B). Next, we compared the expression of TAZ between TRAIL-resistant glioma cells and their parental cell lines. We observed that expression level of TAZ in U87/R and U251/R cells was obviously upregulated at both the mRNA level and protein level compared to the U87 and U251 cells, respectively (Figure 1C). Overexpression of TAZ may be responsible for formation of TRAIL resistance in glioma.

### Expression of TAZ is associated with TRAIL sensitivity in glioma

**Figure 2:**
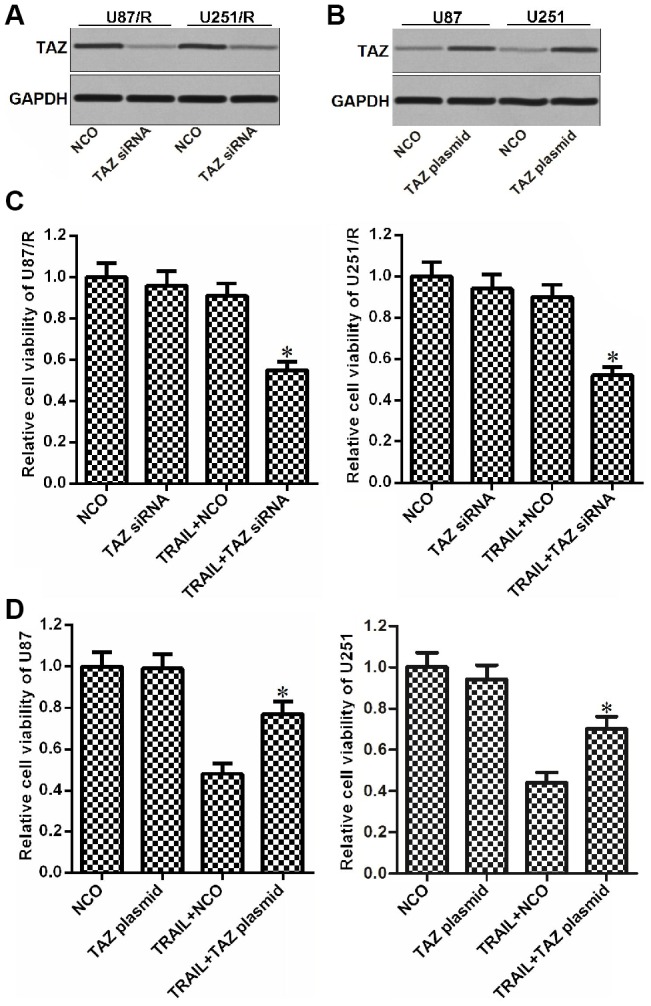
**Role of TAZ in regulating the drug sensitivity of glioma to TRAIL.** (**A**) Effect of TAZ siRNA on decreasing the TAZ expression in U87/R and U251/R cells. (**B**) Effect of TAZ plasmid on increasing the TAZ expression in U87 and U251 cells. (**C**) Sensitization of TAZ siRNA on TRAIL-induced cytotoxicity against U87/R and U251/R. *P<0.05* vs.* TRAIL + NCO group. (**D**) Effect of TAZ plasmid on inducing the TRAIL resistance in U87 and U251. *P<0.05* vs.* TRAIL + NCO group.

To study the role of TAZ in the formation of TRAIL resistance in glioma, we changed the expression of TAZ in U87/R, U251/R (Figure 2A), U87 and U251 cells (Figure 2B) by using TAZ siNRA or plasmid respectively. As TRAIL at the concentration of 10 ng/mL induced slight cytotoxicity to U87/R and U251/R and significant cytotoxicity against U87 and U251, we chose this concentration of TRAIL to study the effect of TAZ on the drug sensitivity in the following experiments. Interestingly, we found that knockdown of TAZ reduced the drug resistance of U87/R and U251/R to TRAIL (Figure 2C). However, overexpression of TAZ in U87 and U251 cells induced their TRAIL resistance (Figure 2D). Taken together, we demonstrated that overexpression of TAZ was responsible for formation of TRAIL resistance in glioma.

### Overexpression of TAZ in TRAIL-resistant glioma cells is caused by downregulation of miR-125b

**Figure 3:**
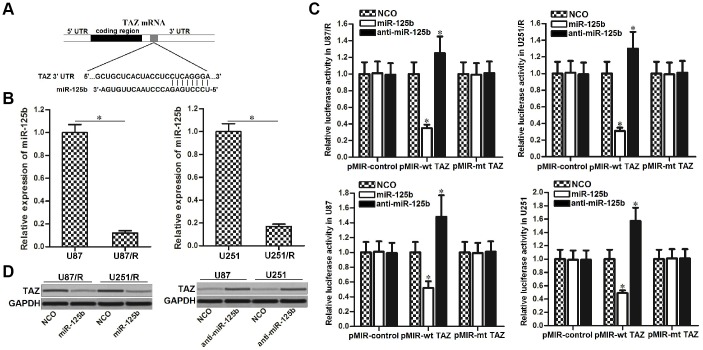
**TAZ is the target of miR-125b in glioma.** (**A**) TargetScan, miRanda and PicTar databases were used to predict the binding site of miR-125b in TAZ 3′ UTR. (**B**) Expression level of miR-125b in U251, U251/R, U87 and U87/R cells. (**C**) U251, U251/R, U87 and U87/R cells were co-transfected with miR-125b mimics or inhibitors and wild-type (wt) or mutant (mt) TAZ 3′ UTR. Luciferase activities were measured by using Dual-Luciferase Reporter Assay System. *P<0.05* vs.* NCO group. (**D**) Western blot analysis of TAZ expression in U251, U251/R, U87 and U87/R cells transfected with miR-125b mimics or inhibitors.

To explore the mechanism by which TAZ was overexpressed in TRAIL-resistant U87 and U251 cells, public miRNA databases of TargetScan, miRanda, and PicTar were used to search the upstream regulator of TAZ. All of these three databases showed that miR-125b contained binding sequence paired with TAZ mRNA 3′ UTR (Figure 3A). After detection of miR-125b expression in U87/R, U251/R, U87 and U251, we observed that expression level of miR-125b was decreased (Figure 3B), whereas expression of TAZ was increased (Figure 1C) in TRAIL-resistant glioma cells. We thus inferred that TAZ was the target of miR-125b in U87/R and U251/R cells. As shown in Figure 3B, the luciferase activity of pMIR-wt TAZ in glioma cells transfected with miR-125b mimics was remarkably lower than cells transfected with NCO. Meanwhile, glioma cells transfected with anti-miR-125b exhibited higher luciferase activity compared to the cells transfected with NCO. However, no significant difference of luciferase activity of pMIR-mt TAZ was observed between the miR-125b group (or anti-miR-125b group) and NCO group (Figure 3C). These results indicated that TAZ was the target of miR-125b in glioma. Results of western blot analysis showed that overexpression of miR-125b in the U87/R and U251/R cells can decrease the protein level of TAZ, meanwhile transfection with anti-miR-125b in U87/R and U251/R cells can increase the expression of TAZ (Figure 3D). We demonstrated that TAZ was the target of miR-125b in glioma, and overexpression of TAZ in TRAIL-resistant glioma cells was caused by downregulation of miR-125b.

### Restore of miR-125b expression reduces the TRAIL resistance in TRAIL-resistant glioma cells

**Figure 4:**
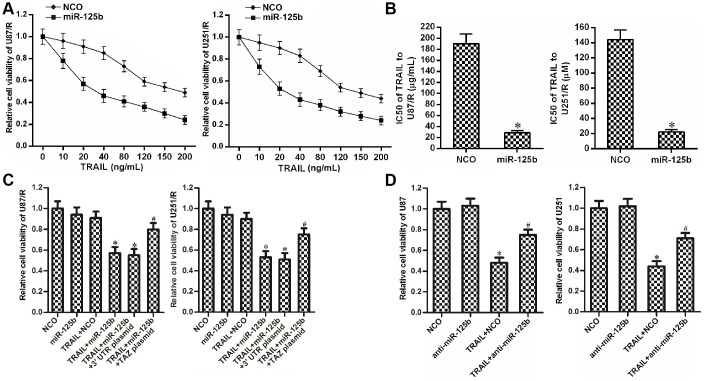
**MiR-125b mimics targets TAZ to attenuate the TRAIL resistance in U251/R and U87/R.** (**A**) MiR-125b mimics increased the drug sensitivity of U251/R and U87/R to TRAIL. (**B**) Effect of miR-125b mimics on reducing the IC50 of TRAIL to U251/R and U87/R. *P<0.05* vs.* NCO group. (**C**) Transfection with TAZ plasmid inhibited the sensitization of miR-125b on TRAIL-induced cytotoxicity against U251/R and U87/R. *P<0.05* vs.* TRAIL + NCO group. ^#^*P*<0.05* vs.* TRAIL + miR-125b group. (**D**) Anti-miR-125b decreased the drug sensitivity of U251 and U87 to TRAIL. *P<0.05* vs.* NCO group. ^#^*P*<0.05* vs.* TRAIL + NCO group.

As TAZ was the target of miR-125b in glioma, we next explored the effect of miR-125b on changing the drug sensitivity of U87/R and U251/R. We observed that overexpression of miR-125b reduced the drug resistance of U87/R and U251/R cells to TRAIL (Figure 4A). We showed that the IC50 of TRAIL to miR-125b-transfected U87/R cells decreased by 77.5% compared to the NCO-transfected U87/R cells. Meanwhile, IC50 of TRAIL to miR-125b-transfected U251/R decreased by 72.1% compared to the NCO-transfected U251/R cells (Figure 4B). These results showed the sensitization of miR-125b on TRAIL-induced cytotoxicity against U87/R and U251/R. However, we found that transfection with TAZ plasmid abolished the effect of miR-125b on reducing the TRAIL resistance of U87/R and U251/R (Figure 4C). Taken together, these results demonstrated that recovery of miR-125b expression can reduce the TRAIL resistance in TRAIL-resistant glioma cells through suppression of TAZ. In addition, despite TRAIL at the concentration of 10 ng/mL induced significant cytotoxicity against routine U87 and U251 cells, knockdown of miR-125b induced significant drug resistance to TRAIL (Figure 4D). These data emphasized that restore of miR-125b expression can reduce the TRAIL resistance in glioma through suppression of TAZ.

### Restore of miR-125b expression targets TAZ to increase the sensitivity of TRAIL-resistant glioma cells to TRAIL-induced mitochondrial apoptosis

**Figure 5:**
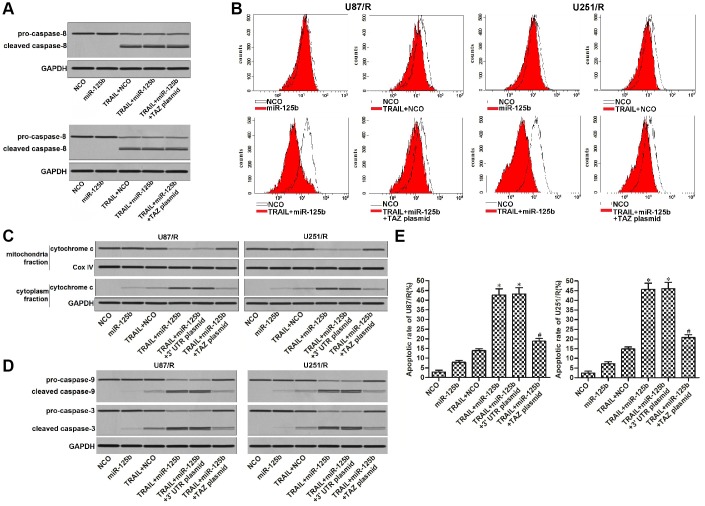
**MiR-125b mimics targets TAZ to enhance the TRAIL-dependent mitochondrial apoptosis in U251/R and U87/R.** (**A**) MiR-125b mimics failed to promote the caspase-8 activation in TRAIL-treated U251/R and U87/R cells. (**B**) MiR-125b mimics targeted TAZ to the enhance the TRAIL-induced damage of mitochondrial membrane potential (MMP) of U251/R and U87/R. (**C**) Protein level of cytochrome c in mitochondria or cytoplasm faction of U251/R and U87/R cells. (**D**) MiR-125b mimics targeted TAZ to the enhance the TRAIL-induced activation of caspase-9 and -3 in U251/R and U87/R cells. (**E**) MiR-125b mimics targeted TAZ to enhance the TRAIL-dependent cell apoptosis of U251/R and U87/R. *P<0.05* vs.* TRAIL + NCO group. ^#^*P*<0.05* vs.* TRAIL + miR-125b group.

We next explored the potential mechanism of miR-125b/TAZ axis on TRAIL treatment. Results of western blot assay showed that transfection with miR-125b and TAZ plasmid can not affect the TRAIL-induced activation of caspase-8 obviously (Figure 5A). We thus inferred that miR-125b/TAZ axis may target the intrinsic apoptosis pathway of glioma cells. Furthermore, TAZ is a mitochondria-related protein that inhibits apoptosis [10, 11], we thus studied the role of miR-125b/TAZ axis in TRAIL-induced mitochondrial apoptosis pathway in U87/R and U251/R. Results of flow cytometry analysis showed that U87/R and U251/R cells were resistant to TRAIL-dependent damage of mitochondrial membrane potential. However, restore of miR-125b enhanced the collapse of mitochondria through suppression of TAZ (Figure 5B). As the results of mitochondria collapse, combination with TRAIL and miR-125b induced release of cytochrome c into the cytoplasm from the damaged mitochondria (Figure 5C). As the downstream of cytochrome c release, caspase-9 and -3 which were markers of intrinsic apoptosis were activated (Figure 5D). Finally, we observed significant apoptotic cell death of U87/R and U251/R cells which were co-treated with TRAIL and miR-125b (Figure 5E). Taken together, we demonstrated that restore of miR-125b expression in TRAIL-resistant glioma cells can increase their sensitivity to TRAIL-induced mitochondrial apoptosis through suppression of TAZ.

### Overexpression of miR-125b increased the sensitivity of TRAIL-resistant glioma tumor to TRAIL in vivo

**Figure 6:**
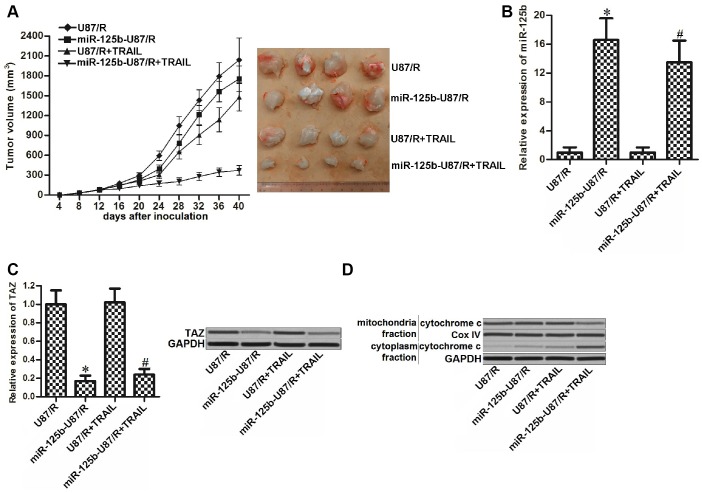
**MiR-125b mimics targets TAZ to attenuate the TRAIL resistance of glioma in vivo.** (**A**) Tumor formation assays were performed with 7 mice per group. The tumor growth was monitored every four days. (**B**) Expression of miR-125b in the purified tumor tissues was detected by qRT-PCR analysis. *P<0.05* vs.* U87/R group. ^#^*P*<0.05* vs.* U87/R + TRAIL group. (**C**) Expression of TAZ in the purified tumor tissues was detected by qRT-PCR and western blot analysis. *P<0.05* vs.* U87/R group. ^#^*P*<0.05* vs.* U87/R + TRAIL group. (**D**) Protein level of cytochrome c in mitochondria or cytoplasm faction of purified tumor tissues.

To test the role of miR-125b/TAZ axis in regulating the sensitivity of TRAIL-resistant glioma tumor to TRAIL in vivo, we inoculated the control U87/R or miR-125b-overexpressed U87/R into the nude mice before treatment with TRAIL. Despite TRAIL showed weak effect on inhibiting the growth of U87/R tumors, we found that miR-125b-overexpressed tumors were more sensitive to TRAIL treatment (Figure 6A). After euthanasia of nude mice followed by purification of tumor tissues, we observed higher expression level of miR-125b (Figure 6B) and lower level of TAZ (Figure 6C) in miR-125b-overexpressed U87/R tumors compared to the control U87/R tumors. Furthermore, we found that under the equal dose of TRAIL treatment, the miR-125b-overexpressed U87/R tumor cells released higher level of cytochrome c compared to the control U87/R tumor cells (Figure 6D). These results indicated that the miR-125b-overexpressed U87/R tumors were more sensitive to TRAIL-induced damage of mitochondria. Combination treatment with miR-125b may be useful for attenuating the drug resistance of glioma to TRAIL in vivo.

## DISCUSSION

Despite TRAIL is a commonly used chemotherapeutic agent in the treatment of glioma, acquired drug resistance is still a problem that should not be ignored in the course of treatment. Recently, studies have reported that TAZ is overexpresed in many cancers [12, 13]. Furthermore, studies have shown that inhibition of TAZ expression restores the tamoxifen sensitivity of drug-resistant breast cancer cells [16]. And in lung cancer, TAZ inhibition increases the sensitivity of cancer cells to cisplatin treatment [17]. Therefore, overexpression of TAZ has been found to be responsible for drug resistance in several cancers, and TAZ may be a potential target for reversing the chemoresistance.

To explore the role of TAZ in the formation of TRAIL resistance in glioma, we established TRAIL-resistant glioma models through gradual exposure to increasing concentrations of TRAIL. In our study, we showed that expression level of TAZ was significantly increased in the established TRAIL-resistant glioma cells. More importantly, we proved that the drug resistance of glioma to TRAIL was partially induced by the overexpression of TAZ. As knockdown of TAZ was found to reduce the TRAIL resistance in the established TRAIL-resistant glioma models, we demonstrated that TAZ can be a potential target during the TRAIL-based chemotherapy of glioma.

MiRNAs are important regulators of human cancer-related genes [18–20]. Studies have demonstrated that formation of acquired drug resistance is closely associated with dysregulation of miRNAs in cancers including glioma [21–23]. Thus, correcting the dysregulation of some specific miRNAs can overcome the chemoresistance of some cancers [24, 25]. MicroRNA-125b (miR-125b) is a tumor suppressor in some cancers. Furthermore, miR-125b is usually used as a sensitizer in the treatment of cancers [26, 27]. In the present study, we found that downregulation of miR-125b was responsible for overexpression of TAZ in the established TRAIL-resistant glioma models. Restore of miR-125b can reduce the TRAIL resistance in glioma through suppression of TAZ.

**Figure 7:**
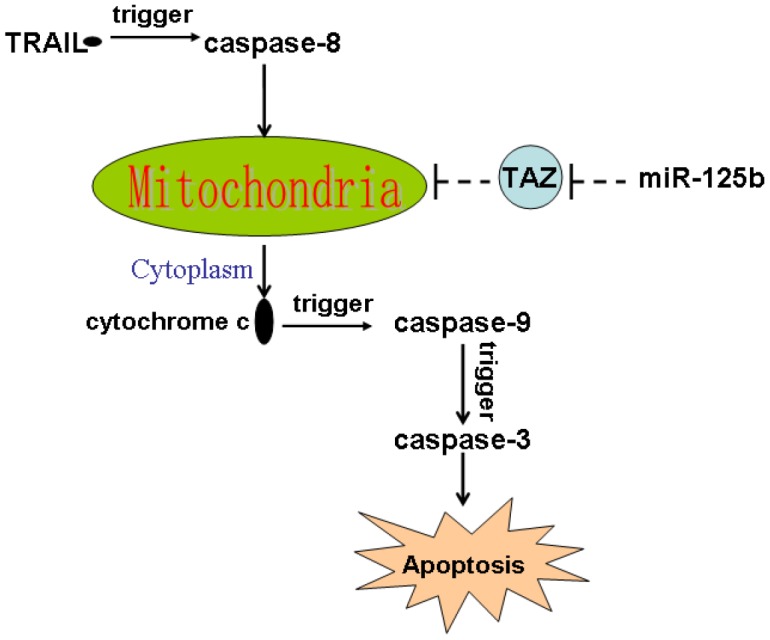
**Schema of the predicted mechanisms implicated in glioma cells response to TRAIL and miR-125b.** Overexpression of miR-125b suppressed the expression of TAZ, and thus promoted the TRAIL-dependent collapse of outer mitochondrial membrane potential and permeability. As the results, combination with miR-125b and TRAIL induced release of cytochrome c into the cytoplasm from the damaged mitochondria. In the presence of cytochrome c, caspase-9 and its downstream caspase-3 was cleaved. Finally, the caspases-dependent apoptosis occurs.

TRAIL induces apoptotic cell death of cancers [28, 29]. It triggers caspase-8 and thus induces extrinsic and intrinsic apoptosis. TRAIL-caused extrinsic apoptosis is induced by activation of caspase-8 directly, whereas the intrinsic apoptosis pathway is required the mediation of mitochondria damage [6, 7]. Additionally, as an important cardiolipin-remodeling enzyme, TAZ is involved in the maintenance of mitochondrial membrane potential which is a key regulator of the mitochondrial apoptosis [10, 30]. In the present study, we tested the effect of miR-125b restore in the established TRAIL-resistant glioma models. However, we failed to observe the effect of miR-125b on TRAIL-induced activation of caspase-8. It suggested that miR-125b may target the intrinsic apoptosis pathway of glioma cells. We then found that overexpression of miR-125b suppressed the expression of TAZ, and thus promoted the TRAIL-dependent collapse of outer mitochondrial membrane potential and permeability. As the results, combination with miR-125b and TRAIL induced release of cytochrome c into the cytoplasm from the damaged mitochondria. Finally, miR-125b promoted caspases-dependent apoptosis through suppression of TAZ in the TRAIL-treated glioma cells (Figure 7).

## MATERIALS AND METHODS

### Cell culture

Human glioma cell lines U87 and U251 were purchased from the Institute of Biochemistry and Cell Biology, Chinese Academy of Sciences (Shanghai, China) and cultured in RPMI-1640 medium (Gibco, Carlsbad, CA, USA). All of the cells were supplemented with 10% fetal bovine serum (FBS) and maintained in a humidifed incubator at 37°C. For acquisition of TRAIL-resistant glioma cell lines, U87 and U251 cells were gradually exposed to increasing concentrations of TRAIL from 0.2 ng/mL to 1 ng/mL. Briefly, U87 and U251 cells were initially treated with 0.2 ng/mL TRAIL for 8 weeks. Subsequently, TRAIL concentration was increased every 3 weeks by 0.2 μg/ml up to a final concentration of 1 ng/mL. The acquired TRAIL-resistant U87 and U251 were named as U87/R and U251/R.

### Total RNAs extraction and quantitative real-time polymerase chain reaction (qRT-PCR)

Total RNAs from cell lines and mice tumor tissues were extracted by using TRIzol reagent (Life Technologies, Carlsbad, CA, USA). Next, cDNAs were synthesized by using the extracted RNAs and One Step PrimeScript miRNA cDNA Synthesis Kit (Takara Bio, Inc., Otsu, Japan). To detect the TAZ mRNA expression, SYBR Premix Ex Taq (TaKaRa) PCR system was used on an ABI StepOne Plus qPCR System (Applied Biosystems, USA). GAPDH was used as an endogenous control. The relative expression of miR-125b was measured by using the same qPCR system. U6 small nuclear RNA (snRNA) was used as an endogenous control.

### Cell transfection

Recombinant pcDNA3.1 plasmid carrying TAZ open reading frame (TAZ plasmid), Recombinant pcDNA3.1 plasmid carrying TAZ 3′ UTR fragment with miR-125b seed region (3′ UTR plasmid), TAZ siRNA (Santa Cruz Biotechnology, Santa Cruz, CA, USA), hsa-miR-125b mimic (5′-AGUGUUCAAUCCCAGAGUCCCU-3′, 50 pmol/ml, GenePharma Co. Ltd, Shanghai, China), anti-hsa-miR-125b (5′-AGGGACUCUGGGAUUGAACACU-3′, 80 pmol/ml, GenePharma Co. Ltd), negative control oligonucleotide (NCO, 5′-CUCAUUCCCAUAUGGUCGCAAG-3′, 50 pmol/ml, GenePharma Co. Ltd) were transfected into cells by using Lipofectamine™ 2000 (Invitrogen, CA, USA) according to the manufacturer’s instructions. Recombinant lentiviral expression vector carrying miR-125b precursor was constructed by GenePharma Co. Ltd. The lentiviruses were transfected into the U87/R cells according to the manufacturer’s instructions.

### Cell viability assay

After 24 h transfection, cells were inoculated into 96-well plates at a density of 1 × 10^4^ cells/well overnight. Next, the adherent cells were treated with different concentrations of TRAIL followed by incubation for 48 h at 37 °C. Subsequently, cells were incubated for 4 h with addition of 20 μl of 5 mg/ml MTT reagent (Sigma-Aldrich, St.Louis, MO, USA) into each well. The optical density (OD) was measured at 570 nm by a microplate reader (Sunrise Microplate Reader, TECAN, Switzerland). Relative cell viability of experimental groups was normalized to the control group. 50% inhibiting concentration (IC50) of TRAIL was calculated according to the cell viability curve.

### Luciferase reporter assay

Fragment of wild type (wt) TAZ 3′ UTR was amplified and subcloned into the pMIR-REPORT™ miRNA Expression Reporter Vector (Thermo Fisher Scientific, Inc, Waltham, MA, USA). The pMIR-REPORT with mutant (mt) TAZ 3′ UTR was created by mutating the seed regions of the miR-125b binding site (CUCAGGGA) by using a site-directed mutagenesis kit (Takara). To perform the luciferase reporter assay, cells were seeded in 24-well plates for 24 h. The miR-125b mimics (or anti-miR-125b, or NCO) and the recombinant pMIR-REPORT were then co-transfected into the cells by using Lipofectamine 2000. 48 h after transfection, luciferase activities were measured by using the Dual-Luciferase Reporter assay system (Promega, Fitchburg, WI, USA) according to the manufacturer’s instructions.

### Western blot analysis

Cells were lysed by using RIPA buffer (Cell Signaling Technologies, Danvers, MA, USA) and the total proteins were collected. A bicinchoninic acid (BCA) protein assay kit (Pierce, Rockford, IL, USA) was then used to quantify the protein concentrations. Next, equal amount of the extracted proteins were separated by 10% sodium dodecyl sulfate polyacrylamide gel electrophoresis (SDS-PAGE) followed by transference to polyvinylidene difluoride (PVDF) membranes (Millipore, Billerica, MA, USA) through electroblotting. The membranes were placed into a plastic bag in the next step and incubated overnight with primary antibodies against human TAZ, GAPDH, cytochrome c, Cox IV, caspase-8, caspase-9 and caspase-3 overnight. After 3 times 5-min washing with phosphate buffer saline (PBS), membranes were probed with horseradish peroxidase-conjugated antibodies. An enhanced chemilu-minescence detection kit (Pierce) was used for signal detection. Cell mitochondria and cytosol was separated by using Mitochondria/Cytosol Fraction Kit (BioVision, Milpitas, CA, USA) according to the manufacturer’s instruction.

### Flow cytometry analysis

Mitochondrial membrane potential (MMP) and cell apoptosis were detected by flow cytometry. After treatment, cells were harvested and washed twice with PBS. To detect the mitochondrial membrane potential (MMP), cells were resuspended in PBS and stained with 5,5′,6,6′-Tetrachloro-1,1′,3,3′-tetraethyl imidacarbo cyanine iodide (JC-1, Molecular Probes; Waltham, MA, USA) as an indicator. To measure the cell apoptotic rate, Annexin V-FITC Apoptosis Detection Kit (Sigma-Aldrich) was used according to their manufacturer’s instruction. The Annexin V-positive cells were considered as the apoptotic cells.

### In vivo experiment

5×10^6^ lentiviruse-transfected U87/R cells were inoculated subcutaneously into the BALB/c nude mice (Shanghai Super-B&K Laboratory Animal Corp., Ltd., Shanghai, China). TRAIL (5 mg/kg) were administrated by intraperitoneal injection twice a week after xenografts reached 0.5 cm in diameter. Tumor volume was measured every 4 days. All mice were sacrificed after 40 days. Tumor volume was monitored by measuring the length (L) and width (W) with calipers and calculated using the following formula: tumor volume = L × W^2^/ 2. The animal care and experimental protocols were approved by the Animal Care Committee of The Second Xiangya Hospital, Central South University.

### Statistical analysis

All data are represented as the mean ± standard deviation (SD). Statistical analyses were conducted by using SPSS 15.0 software and illustration data were performed by GraphPad Prism. Two-tailed Student’s t-tests were used to estimate the statistical differences between two groups. One-way analysis of variance (ANOVA) and Bonferroni’s post hoc test were used to determine the differences between three or more groups. *P*<0.05 was considered statistically significant.

## CONCLUSIONS

Overexpression of TAZ partially induced drug resistance of glioma to TRAIL. Furthermore, we proved that absence of miR-125b was responsible for the overexpression of TAZ in the TRAIL-resistant glioma cells. In the apoptosis pathway caused by TRAIL treatment, miR-125b targeted TAZ to promote the collapse of mitochondria. Despite further studies are still required to determine the clinical prospect, miR-125b/TAZ axis may be a potential target to reverse the TRAIL resistance in glioma.
